# Intestinal Antisepsis in Dyspepsia

**Published:** 1891-03-14

**Authors:** 


### The Practitioner's Mirror and Retrospect.

[*The Editor will be glad to receive offers of co-operation and contributions from members of the profession. All letters should be addressed to* The Editor, The Lodge, Porchester Square, London, W.]

### MEDICINE.

### INTESTINAL ANTISEPSIS IN DYSPEPSIA.

Until a comparatively recent period it was almost universally held that the sole action of hydrochloric acid in the gastric juice was to enable the pepsin to act on the proteids of the food, it being inert in any but a distinctly acid medium. But inasmuch as the food in passing into the small intestine was subjected to the still more effective powers of the pancreatic secretion acting in an alkaline medium, a great waste of energy was involved in secreting such an unnecessary acid if this theory was correct.

Drs. Kurlow and Wagner have recently investigated the influence of gastric juice on pathogenic organisms, and they found that only the most prolific microbes, such as tubercle bacilli, the bacilli of anthrax, and perhaps the staphylococci can continue to exist in the normal gastric secretion; all others are destroyed by this powerful germicidal agent in less than half an hour. Their observations led them to believe that constant or specific microbes do not exist in the stomach, and those which enter it with the food are only very temporary and accidental residents. Bunge, in his lectures on "Physiological and Pathological Chemistry," reminds us that the antiseptic action of the gastric juices was noticed by Spallanzani more than a hundred years ago. Spallanzani found that decomposition was prevented in meat for several days if it was moistened with gastric juice, whereas if water was used under similar circumstances, decomposition and an unbearable putrid odour soon developed. A snake had swallowed a lizard. After sixteen days Spallanzai opened the stomach, but although the lizard was half digested, it gave no odour of decomposition. But not only does gastric juice prevent decomposition, it stops putrefaction when it has already begun. Spallanzani found that when decomposing meat was introduced into the stomachs of various animals it lost its putrefactive character after a time, and particularly its putrid odour. The same process, of course, is constantly taking place in the human stomach whenever high game or tainted meat is eaten and digested, and thus under normal circumstances putrefactive decomposition of the food scarcely occurs at all. Very careful experiments have been made by Miguel, Seaber, and others to determine the amount of free hydrochloric acid necessary to prevent decomposition taking place in foods, showing that from 0·3 to 0·4 per cent. is sufficient. Bunge further recalls the fact that in Hoppe-Seyler's laboratory, the free acid contained in the undiluted gastric juice, obtained from the stomach of a man by means of the stomach pump, was determined, and that 0·3 per cent. of hydrochloric acid was found. Thus we have the striking result that the quantity of free hydrochloric acid present in the gastric juice exactly corresponds to the quantity which is necessary to prevent the development of putrefactive organisms.

These experiments and observations have, of course, a very great practical interest in connection with some forms of dyspepsia. Normal digestion is a process of fermentation taking place in a suitable antiseptic solution, the physiological ferments belonging to the class of unorganized ferments, while many forms of pathological digestion are the result of fermentation processes, these pathological ferments being different organized micro-organisms.

Now Dr. W. S. Christopher has recently shown how many organized ferments require that the substances which they are to act upon shall first have been modified by some other ferment, either soluble or organized, before they can exert their peculiar action. For instance, the yeast plant cannot ferment cane sugar directly, while it can induce the alcoholic fermentation in the glucoses. The first part of the process of alcoholic fermentation by the yeast plant is the inversion of the sugar; that is, it is transformed from cane sugar into dextrose and levulose, two glucoses. This inversion of cane sugar is accomplished by a soluble ferment which accompanies and is probably produced by the yeast plant itself. At all events, writes Dr. Christopher, its action precedes the alcoholic fermentation induced by the plant. Now, in the gastric digestion of proteids the process ends with the formation of peptones, but in the pancreatic digestion of proteids the decomposition of the albuminoid molecule is more profound, and we find, in addition to the peptones, leucine, tyrosine, hypoxanthin, aspartic acid, and glycocol. With these products the fermenting action of the pancreatic juice probably ends, but this action has paved the way for the growth of micro-organisms, which flourish among the products of the tryptic digestion, and as the result of their action upon these products there are produced such bodies as indol, scatol, phenol, fatty acids, ammonia, hydrogen, sulphuretted hydrogen, carbonic acid, and ptomaines. And Dr. Christopher goes on to point out that since this putrefactive process is impressed upon the products of digestion, and is not a change produced in the original food stuff itself, but rather a continuation of the decomposition processes set up in the albuminoid molecule by the digestive ferment proper, it seemed to him that the term *super-digestion* fitly characterizes it, and that certainly the term *indigestion* is eminently improper and misleading.

Of all these bodies arising from super-digestion of proteids the most important are the ptomaines. Dujardin-Beaumetz attributes to them many of the most serious symptoms in neurasthenic dyspepsia. It is the function of the liver, as proved by Schiff, to arrest and destroy these ptomaines, when, after absorption, they are carried to the liver. The ptomaines are alkaloids, and these animal alkaloids, produced by a process possibly analogous to the process of formation of vegetable alkaloids, like the latter, produce their effects by their action on the nervous centres, giving rise to the group of symptoms termed biliousness. Flatulence and acidity, producing heart-burn, are almost invariable accompaniments of such fermentative decompositions. The flatulence is due to the carbonic acid gas liberated, often from lactic acid, splitting up into butyric acid, water, and carbonic acid gas, or sometimes from glucose undergoing fermentation and being decomposed into alcohol and carbonic acid gas. It has of late become a fashion to extol intestinal antisepsis as the remedy for such forms of fermentative dyspepsia, and rightly too. But it may well be questioned whether the introduction of intestinal antiseptics is to be desired. Hydronaphthol, β. naphthol, α naphthol, salicylate of bismuth, and many other excellent antiseptics have all received their advocates and share of praise. Certainly they will act by preventing the action of these organisms which produce fermentation changes. But we have indicated how important a part is played by normal gastric juice as an antiseptic, and we would rather urge the adoption of therapeutic measures which seek to promote intestinal antisepsis by restoring the deranged functions of the gastric mucous membrane.

If it can be tolerated—that is, if the stomach is not in too irritable a condition—nux vomica in combination with a small quantity of the dilute hydrochloric acid would seem to fulfil these conditions. If, however, we have to deal with a case in which the prolonged action of these fermentation processes has set up a great amount of gastric irritability, the salicylate of bismuth, given in three or four grain doses in suitable combination, will be better borne until the treatment more in accordance with physiological principles can be adopted.

